# The effects of repeated automated plasmapheresis in goats (*Capra hircus*) in response to vaccination with purified influenza hemagglutinin proteins

**DOI:** 10.1371/journal.pone.0195903

**Published:** 2018-06-13

**Authors:** Willie D. Taylor, Gregory L. Langham, James L. Weed, Thomas Rowe, Wei Song, Kristin A. Isenberg, Xiyan Xu, David E. Wentworth, George Lathrop, Nathaniel Powell

**Affiliations:** 1 Comparative Medicine Branch, Division of Scientific Resources, National Center for Emerging and Zoonotic Infectious Diseases, Centers for Disease Control and Prevention, Atlanta, Georgia, United States of America; 2 Virology Surveillance and Diagnosis Branch, Influenza Division, National Center For Immunization And Respiratory Diseases, Centers for Disease Control and Prevention, Atlanta, Georgia, United States of America; 3 Program Evaluation Branch, Division Of HIV/AIDS Prevention-Intervention, National Center for HIV/AIDS, Viral Hepatitis, STD, & TB Prevention, Centers for Disease Control and Prevention, Atlanta, Georgia, United States of America; 4 Battelle, Atlanta, Georgia, United States of America; Icahn School of Medicine at Mount Sinai, UNITED STATES

## Abstract

Seasonal influenza is a contagious respiratory illness that annually affects millions of people worldwide. To identify currently circulating influenza virus subtypes, the Centers for Disease Control and Prevention’s International Reagent Resource distributes the World Health Organization (WHO) influenza reagent kits, which are used globally by testing laboratories for influenza surveillance. The data generated by the kits aid in strain selection for the influenza vaccine each season. The use of animals to produce high quality and quantities of antibodies is critical to the production of these kits. In this study, we assessed the effects and efficacy of repeated sampling from automated plasmapheresis in goats. Analysis of blood samples demonstrated that repeated automated plasmapheresis procedures did not adversely affect the immediate or long-term health of goats. Further, our results indicate that repeated plasmapheresis in goats was capable of generating 2 liters of antibody-rich plasma per goat per week. This volume is sufficient to produce enough WHO influenza kits to conduct over 1 million tests. Thus, we have shown that the rapid production of plasma in goats can positively impact the public health preparedness and response to influenza.

## Introduction

Influenza is a contagious respiratory illness that has resulted in over 22 million illnesses, 425,000 hospitalizations, and an estimated 34,000 deaths annually since 2010[1–5]. Each year, on average, 5 to 20% of the U.S. population contracts influenza, costing an estimated $10.4 billion in medical expenses and $16.3 billion in lost earnings[6]. Global surveillance to protect these populations from seasonal influenza outbreaks requires extensive monitoring of circulating influenza viruses.

Currently, two subtypes of influenza viruses A viruses, A(H1N1)pdm09 and A(H3N2), and two lineages of influenza B viruses (B/Victoria and B/Yamagata lineage) are co-circulating worldwide in each influenza season. However, the prevalence of these four seasonal influenza virus groups varies each year. Circulation and antigenic changes of influenza viruses are monitored worldwide by the World Health Organization (WHO) Global Influenza Surveillance and Respond System (GISRS) to identify the currently circulating viruses and to assist the WHO to make recommendations for composition of influenza vaccine for the following influenza season. Selection of influenza virus vaccine candidates, which antigenically represent the majority of circulating virus strains, is critically important to annual vaccine production. The Hemagglutinin Inhibition (HI) assay is the primary serological method used to identify and antigenically characterize influenza viruses. This test measures how effectively a known reference antisera inhibits an unknown influenza viruses ability to agglutinate red blood cells, thereby assessing antigenic charcteristics. Health official and scientists can determine what control measure can be used for the control of influenza.

In the past three decades, the CDC has produced large amounts of hyperimmune reference antisera, primarily in sheep, to manufacture the WHO Influenza Reagent Kits. This kit provides reagents for identification of all 4 type/subtype circulating seasonal influenza viruses by HI assays for laboratories of GISRS for influenza surveillance. Historically, reference antisera generated from sheep by repeated inoculation with influenza antigens often yielded increasingly high levels of cross-reactivity to unrelated influenza virus subtypes. Although immunized ferrets yield strain specific antisera, it proved to be costly and inefficient because infected ferrets tend to yield less than 25 ml of antisera per animal. In addition, WHO Influenza Kit reagents must be evaluated and updated every year ensuring its sensitivity and specificity. Since public health institutions around the world rely on timely delivery of the WHO Reference Kit, these challenges led us to evaluate goats for efficiently generating sizable volumes of influenza-specific plasma.

Previous studies by Klages[7] showed that a single goat is capable of producing 2000 mL of antibody-rich plasma per month without demonstrating adverse clinical effects. To obtain plasma from a large animal donor, plasmapheresis is traditionally performed manually by removing whole blood; allowing the plasma to separate from the cellular mass; extracting the plasma component; and returning the re-suspended red blood cells to the donor. This sedimentation technique is simple, but has many disadvantages, including the potential for bacterial contamination and increased cellular components in the plasma, which may create hypersensitivity in recipients. Nearly four decades ago, the introduction of automated in-line blood cell separators revolutionized the plasmapheresis process[7]. These in-line automated plasmapheresis units (APU) were initially developed for plasma collection in human healthcare operations and are now used in the research setting to safely and efficiently collect immunoglobulin-rich plasma for use as bio-reagents. Continuous-flow plasmapheresis allows for rapid and sterile separation of blood into its component parts, collection of the desired amount of plasma, and return of the cellular components to the donor. Feige et al [8] looked at various plasma collection protocols using APU’s and evaluated the associated effects on clinical, hematological, and coagulation variables in horses. Klages[7] demonstrated that repeated AP in goats was clinically safe and an improvement to current antibody recovery techniques, but did not measure AP’s impact on coagulation. This research provided the impetus for the current study.

Herein, we hypothesized that there would be no differences between serial physiological samples obtained from immunized goats via repeated automated plasmapheresis in a condensed time period. These potential perturbations in sample quality or consistency have not been observed in humans undergoing similar repeated plasmapheresis procedures[9–14]. The focus of this study was to refine our antibody-recovery process by using an automated system. Moreover, we evaluated the effect of decreased time required to produce large volumes of antibody-rich plasma; reduction of the number of animals required to produce HI test kits; replacing a terminal procedure with a survival procedure; and monitored the effects of repeated AP on hematological parameters in goats.

## Materials and methods

### Ethics statement

Research was conducted under an approved Center for Disease Control and Prevention Institutional Animal Care and Use Committee protocol in compliance with the Animal Welfare Act[15] and other federal statutes and regulations relating to animals and experiments involving animals and adhered to principles stated in the *Guide for the Care and Use of Laboratory Animals*[16]. The CDC is accredited by the Association for the Assessment and Accreditation of Laboratory Animal Care, International.

### Animals

Six female 15-month old goats (five Lamancha and one Nubian, Latimer Luck Acres, Watkinsville, GA) averaging 33 kg were used in this study. All animals underwent an annual screening regimen at the vendor and were serum-negative for brucellosis, tuberculosis, and scrapie. Each goat was vaccinated against *Clostridium perfringens* Types C and D and tetanus (Bar Vac CD-T Cattle, Sheep and Goat Vaccine Boehringer Ingelheim Vetmedica, Inc. Duluth, GA, USA) in addition to an annual screen for caprine arthritis encephalitis virus. Baseline serum chemistries, complete blood counts, fibrinogen levels, and clotting times were established for each animal during the 14-day quarantine period. Following quarantine, a serum sample was drawn and tested for antibodies to currently circulating seasonal influenza viruses by hemagglutinin inhibition assay. Goats were able to freely access water and mixed-grass pastures, as well as barns for protection against inclement weather. In addition, goats were provided hay, mineral and poloxalene-medicated blocks (Sweetlix® Bloat Guard®, Ridley Block Operations, Mankato, MN, USA). Two to three times per week, a ruminant supplemental diet was given *ad libitum* at a rate of 1–2% body weight per day (Rumilab®, 5508 Diet Lab Diet, St. Louis, MO, USA).

### Viruses and immunization

Seasonal, human influenza viruses were amplified in embryonated chicken eggs[4] and hemagglutinin (HA) of the viruses were extracted using bromelain (Brom-HA) [17]. In this study, we utilized representative viruses (B/Texas/02/2013 and B/Brisbane/06/2008) of the B/Victoria lineage of influenza. For generation of a broadly covering immune response generally 2–3 HA’s/subtype are used [prime (N = 1), and, boost (N = 1 or 2)] to cover the vaccine strain and representative antigenic groups within a subtype. In this study we used B/Texas/02/2013 as the priming strain and B/Brisbane/60/2008 as the boosting strain. The initial injection (Group 2 N = 3) for antibody production consisted of 100 μg HA mixed at a 1:1 ratio with adjuvant (TiterMax Gold^TM^, TiterMax USA Inc., Norcross, GA, USA) administered intramuscularly (IM), followed by subsequent biweekly boosts of 30 μg HA with adjuvant one month following primary inoculation. Intramuscular injections were given in alternating gluteal muscles. Blood samples were drawn weekly to assess antibody levels. Once antibody levels reached an HI titer of ≥1:160, the animal was placed on the plasmapheresis schedule. Control goats (Group 1 N = 3) were administered adjuvant only following the above schedule.

### Restraint

A Panepinto sling® (Panepinto & Associates, Masonville, CO, USA) restraint device with a customized hammock was used for the plasmapheresis procedure. The Panepinto sling unit was chosen to facilitate restraint of animals for plasma collection[18]. A small-ruminant head harness (Sydell. Burbank, SD, USA) was attached to one end of the sling. This facilitated positioning of the animal’s head and neck for venous access. This allowed the animal to maintain a relatively normal position during the plasmapheresis procedure. In addition, a cloth was placed over the animals eyes to decrease visual stimulation. Animals were continuously monitored throughout the plasma collection cycle.

### Plasmapheresis

On the day of plasmapheresis, the goats were visually examined, placed into the sling, and elevated to access the jugular vein. Hair was shaved from the selected jugular groove, prior to aseptic preparation with an iodine/chlorohexidine scrub and isopropyl alcohol. Each goat was sedated with xylazine 0.02 mg/kg IV (AKORN Animal Health Lake Forest, IL. USA), and an intravenous catheter (14–18 gauge) was placed to draw blood for serum chemistries and hematology (pre-plasmapheresis samples) prior to the animal being connected to the APU. During subsequent procedures, catheters were placed in alternating jugular veins to reduce trauma. Plasma was collected in a 1000 mL Plasmalink^™^ bottle with a locking Luer adapter (Fenwal International Inc. Haina, Dominican Republic). The APU continually removed whole blood during each collection cycle by separating the sample into acellular plasma and cellular blood components until approximately 120 mL of predominately red cells was collected. The re-suspended cellular components were then diverted back into the animal before beginning another collection cycle. The plasma was directed continually into the 1000 mL Plasmalink™ bottle. The collection and reinfusion cycle continued until it reached the pre-programmed volume of 500 mL plasma. When sample collection was complete, 500 mL of 0.9% saline solution was administered to offset the plasma volume removed. After the saline infusion, blood was drawn for serum chemistries and hematology (post-plasmapheresis samples). Prior to removing the IV catheter, a 4 x 4 inch gauze pad was placed over the venipuncture site and held in place until hemostasis was achieved. Plasma was labeled and stored in a -70° C freezer. The animal was then moved to a barn stall for post-procedural monitoring. Here the goat was visually observed for signs of distress or changes in behavior every hour for approximately 2 to 4 h before being returned to the pasture. At 24 and 48 h after plasmapheresis, additional blood samples were collected to monitor serum chemistry and hematology. Subsequently, two goats (one from each group) underwent plasmapheresis at 24, 48, or 168 hour- intervals, totaling 4 sessions. All serum chemistries, complete blood counts, and clotting times were evaluated by Antech Laboratories (Smyrna, GA. USA) Note: All animals are currently still on study.

### Hemagglutination inhibition assay

The hemagglutination inhibition (HI) assay was performed using WHO established procedures[4]. Briefly, goat sera was treated with receptor destroying enzyme (RDE) (Denka Seiken. Nigata, Japan) to remove non-specific inhibitors. Viruses were standardized to 4 HA units/25 μL and tested against two-fold serial dilutions of RDE treated goat antisera in 96-well v-bottomed plates. After a 15 min incubation, 50 μL of 0.5% turkey red blood cells (TKRBCs) were added to each well and plates were incubated at room temperature (22 to 25°C) for approximately 30 min to allow agglutination of the TKRBCs. The plates were tilted, and hemagglutination inhibition titers (HI) were recorded. Titers were reported as the reciprocal of the highest dilution of antisera demonstrating complete inhibition of virus agglutination.

### Data analysis

Data were normally distributed and met all assumptions of ANOVA. Data were analyzed using repeated measures of ANOVA within groups. Tukey posthoc adjustments for multiple comparisons were applied where appropriate[19, 20]. Analyses were conducted using SAS statistical software (Version 9.4, SAS Institute, Cary, NC, USA). P values <0.05 indicated statistical significance.

## Results

**Fig 1:**
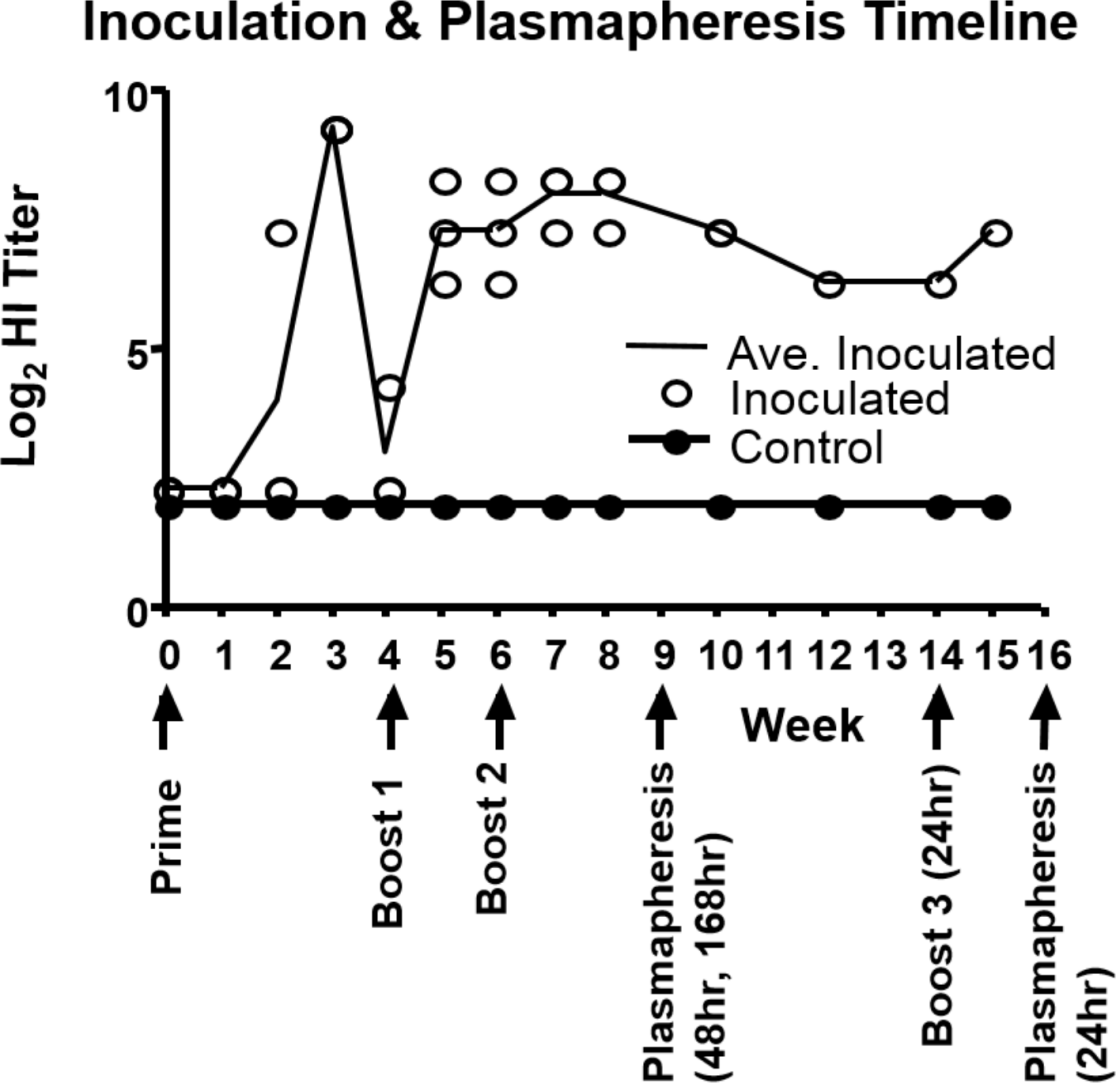
Inoculation & plasmapheresis timeline. Kinetics of anti-influenza antibodies in goats following vaccination. HI data for antibody responses to B/Brisbane/60/2008 following prime/boost of goats. Data show average (log_2_) titers, reciprocal of last dilution of antisera resulting in complete inhibition of red blood cell agglutination by virus, of individual goats (open circles), and sham goats (closed circles) and average HI titer (line). The prime/boost schedule and when plasmapheresis was initiated is indicated by arrows.

To evaluate the medical effects of AP on goats and determine the optimal AP frequency for the generation of influenza-specific plasma, we inoculated and plasmapheresed six goats over a 16 week period. At day 0, three experimental goats were primed with purified influenza B/Texas/2/2013 virus Brom-HA protein plus adjuvant and three control goats were inoculated with adjuvant only. Goats were boosted with B/Brisbane/60/2008 Brom-HA protein at four and six weeks post prime and one goat received an additional boost at 14 weeks to increase serum antibody titers. When subtype-specific HI titers in vaccinated animals were ≥1:160, an experimental and control animal pair was plasmapheresed 4 times at either 24, 48, or 168 hour intervals (Fig 1).

Blood samples were obtained from all goats before and after each of four plasma collection sessions to determine if the AP procedure adversely affected the health of the goats. Each sample was analyzed for clotting times, complete blood counts, and serum chemistry profiles (Table 1).

**Table 1:** Comparison of normal blood chemistries with experimentally obtained values.

Parameters		Group 1 (*n* = 24)			Group 2 (*n* = 23)		
	Normal ranges	Pre (*n* = 12)	Post (*n* = 12)	F test	Pre (*n* = 12)	Post (*n* = 11)	F test
PT time	9.0–14.8 sec	11.66	13.53^b^	14.19	11.28	12.87^b^	9.90
PTT time	28.4–37.6 sec	22.76	26.43^b^	13.64	25.55	28.65^b^	9.47
Fibrinogen	100–400 mg/dl	201.92	131.52^c^	19.31	187.17	128.09^b^	9.50
TP	6.5–8.8 g/dl	5.79	4.03^c^	22.85	5.43	4.15^b^	10.54
Albumin	3.0–4.0 g/dl	2.35	1.61^c^	21.44	2.20	1.70^b^	10.26
Globulin	2.5–5.8 g/dl	3.44	2.42^c^	19.97	3.23	2.45^b^	9.56
ALT	15–50 IU/L	10.83	8.00^b^	11.84	12.50	9.55^b^	1.09
ALP	63–263 IU/L	132.75	97.50^b^	10.23	464.17	331.36^d^	0.93
Urea	5–24 mg/dl	18.67	17.00^d^	1.04	20.42	19.64^d^	0.12
Creatinine	0.6–1.6 mg/dl	0.54	0.46^d^	4.26	0.53	0.47^d^	2.16
Glucose	50–90 mg/dl	88.58	127.08^a^	6.76	88.83	140.09^b^	11.81
WBC	7.0–15.0 10^3 μl	11.90	9.27^a^	6.82	12.24	8.40^b^	10.55
RBC	8.0–18.0 10^6 μl	14.21	11.85^a^	7.88	15.45	11.85^a^	7.55
Hemoglobin	8.0–14.0 g/dl	7.73	6.48^b^	8.74	8.70	6.70^a^	6.97
Hematocrit	19–38%	23.08	22.50^d^	0.04	29.33	19.27^a^	5.11
Platelet Ct	120–550 10^3 μl	892.50	638.75^a^	5.51	479.42	334.82^b^	8.75

*n* = 24 is samples from Group 1 (Control)

*n* = 23 is samples from Group 2 (Experimental)

^a^*P* value for Pre and Post is < 0.05 but not < 0.01

^b^*P* value for Pre and Post is < 0.01 but not < 0.001

^c^*P* value for Pre and Post is < 0.001

^d^*P* value is not significant

TP = total protein

Data analysis revealed there were significant differences between pre- and post-experiment samples within each group. In Group 1 (control), there were significant differences in all variables tested except for Urea concentration (p = 0.3201, ns), Creatinine (p = 0.0524, ns), and Hematocrit values (p = 0.8518, ns). In Group 2 (experimental) there were significant differences in all variables tested except in ALP (p = 0.3463, ns), Urea concentration (p = 0.3463, ns), and Creatinine (p = 0.1579, ns). The significant differences and number of samples represented within each group are presented in Table 1.

In sham inoculated control and experimental animals, fibrinogen, platelet, albumin, globulin, creatinine, urea, and hemoglobin blood proteins levels marginally declined, but remained above levels correlated to normal reference ranges. As expected, these results were accompanied by slightly increased, but normal, PT and PTT times in both animal groups. Both ALP and ALT levels also dropped following AP to levels consistent with regular liver, gall bladder, and bone function. Similarly, hematocrit, WBC, and RBC levels declined in response to AP, but did not indicate a clinically relevant leukopenia or anemia. Altogether, these results indicate that AP, and the removal of 2000 ml of plasma, does not adversely affect the health of goats.

**Fig 2:**
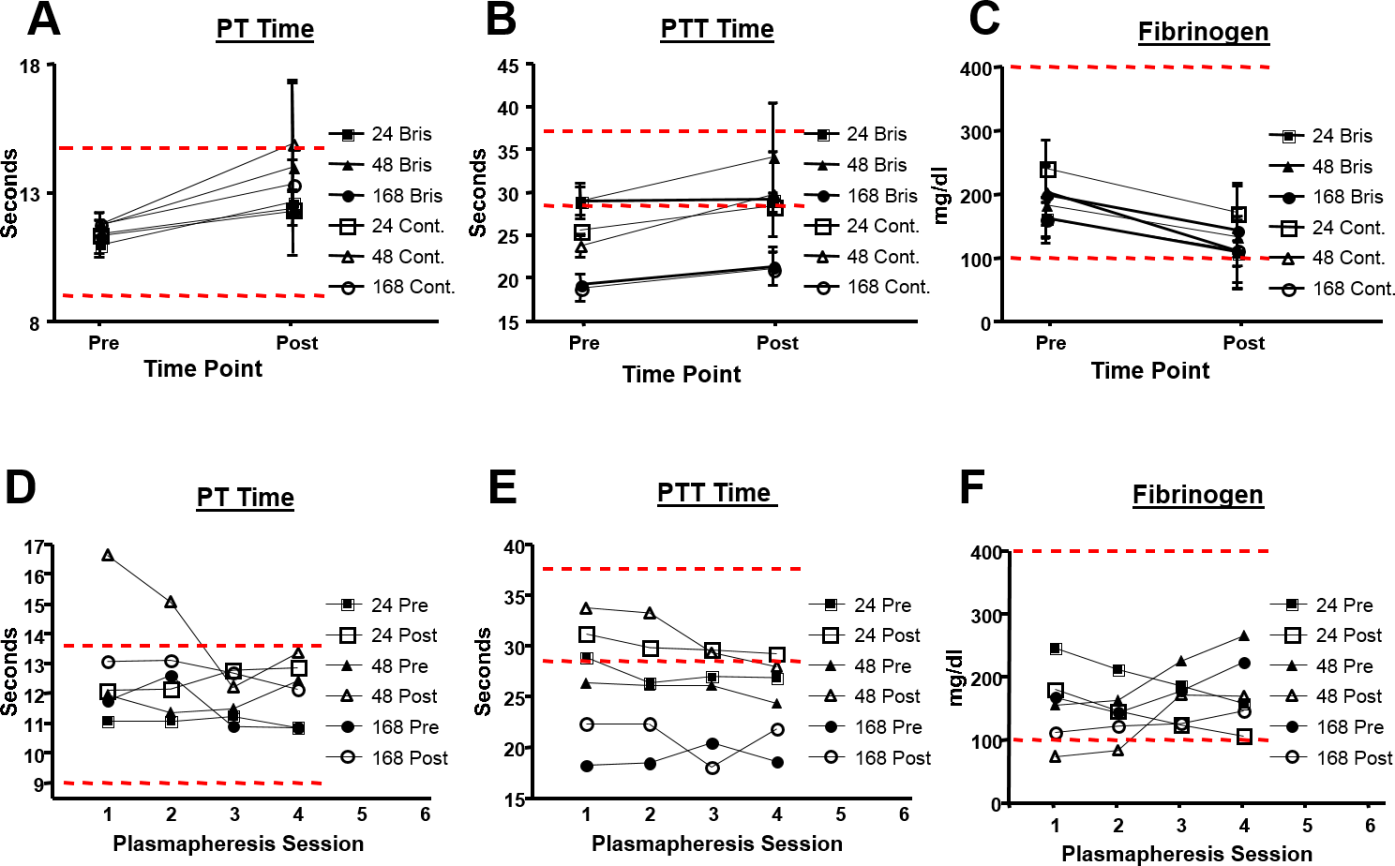
Effects of multiple repeated plasmapheresis on clotting times. Red dotted lines represent normal reference ranges (upper and lower limits).

During each of the four plasma collection procedures, the APU cycled an average of 1716.95 mL of whole blood through the machine over approximately 43 min to yield 500 mL plasma. To more closely evaluate if the AP procedure resulted in clinical abnormalities, we analyzed the average PT times, PTT times, and fibrinogen levels in individual goats before and after the four AP procedures (Fig 2A–2C).

Although the average PT and PTT times increased flowing AP, the average pre and post AP test values did not exceed the upper normal clinical range. Similarly, fibrinogen levels dropped in response to AP, but did not fall below normal expected values. In all cases, these clinical test values returned to baseline prior to the subsequent AP procedure. To determine if repeated rounds of AP adversely affected blood clotting, we longitudinally analyzed the same factors after each AP procedure (Fig 2D–2F). In one goat, PT times exceeded the normal clinical range and fibrinogen levels were below the normal clinical range after the first AP collection and the second AP collection 48 hours later. PTT times and other blood work at these time points were normal and PT times and fibrinogen levels normalized through the third and fourth AP procedure. All other goats showed no adverse effects as a result of repeated AP. The AP procedure was well tolerated by each animal and no signs of other blood abnormalities were noted in any individual animal regardless of the collection frequency. In addition, all individual goats were clinically normal based on assessment of animal’s pre- and post-procedural behavior; activity; weight; clinical pathology; and physical examination. Altogether, this data suggests that the individual AP procedure and four repeated AP procedures do not create unsafe conditions for goats.

**Fig 3:**
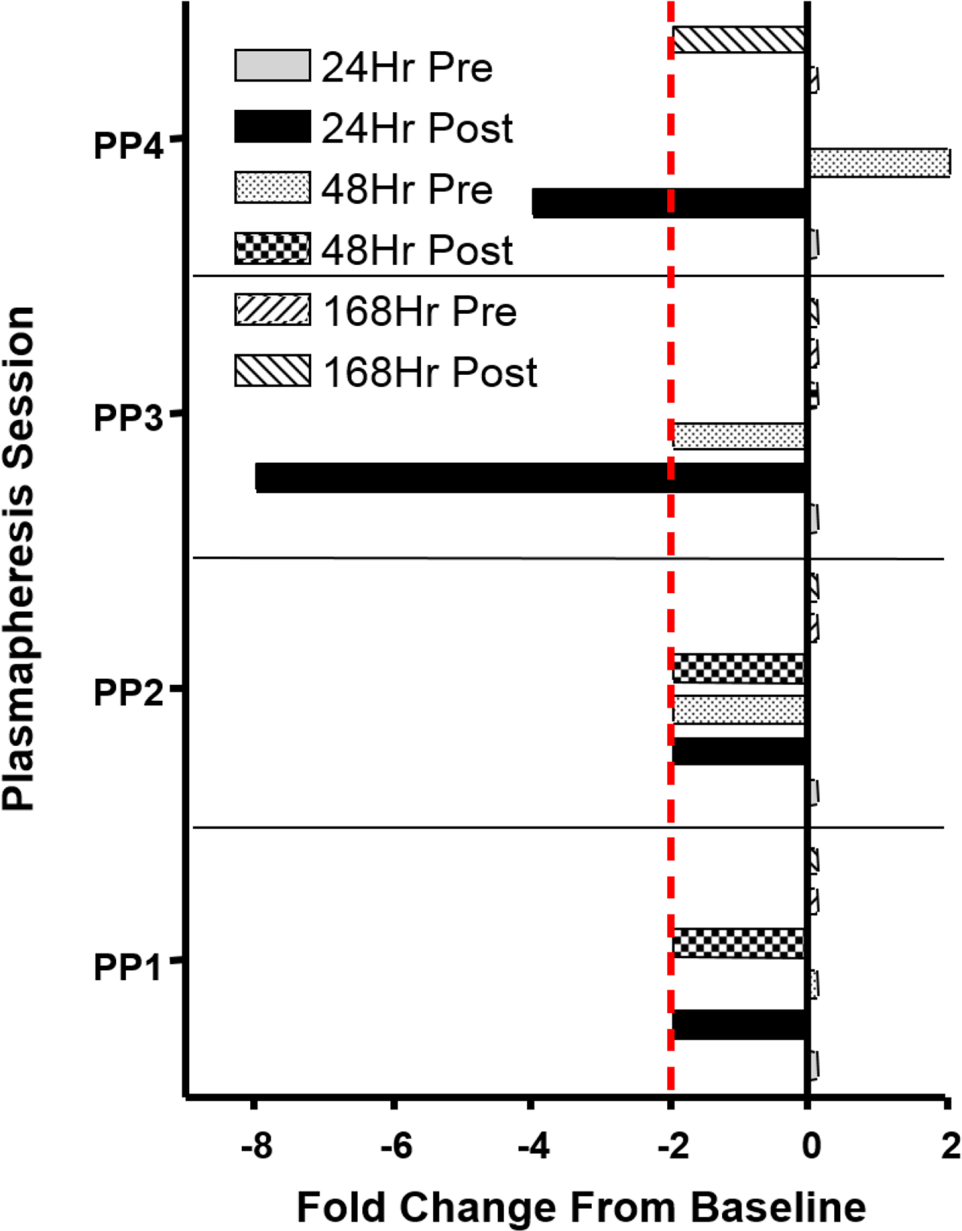
Fold changes in HI antibody response from initial titer.

To determine the optimal AP interval for generating high polyclonal antibody yields, we assayed HI antibody titers in goat plasma which were plasmapheresed at 24, 48, and 168 hours intervals (Fig 3).

Goats that underwent AP at 48 and 168 hour intervals did not show significant decreases in serum titers after four AP collections. In contrast, goats that underwent AP at 24 hour intervals showed reduced serum titers after the third and fourth AP collection (Fold Change ≤ -2). This data shows that 48 hour AP intervals were optimal for maximizing antibody yield, allowing time for goats to replenish serum antibody levels.

## Discussion

The goal of the present study was to evaluate the effects of multiple AP collection procedures on goats inoculated with influenza HA antigen and to determine the optimal AP collection procedure for generating high titer plasma. We were also interested in evaluating whether our procedures resulted in any changes in clotting times PT, PTT, fibrinogen, TP, albumin, globulin, ALT, glucose, WBC, RBC, hemoglobin, and platelet count before and after AP. These values largely remained within the normal ranges and always returned to baseline prior to each subsequent procedure (Table 1 and Fig 2). These results were anticipated because the AP procedure removed approximately 500 ml of plasma at each collection point (Fig 3) and minor changes to blood parameters due to blood loss can self-correct over time[21]. Animals were not negatively affected by the repeated sampling procedures as they exhibited no abnormalities in other blood tests, behavior, activity, or weight. However, two individual animals showed specific-biochemical values outside the normal laboratory ranges for ALP and platelets but neither of these animals demonstrated any significant variation from their established normal prior to the course of study. This observation may be due to osteoblast function of growing bone[22] or a hereditary abnormality, a secondary effect of inflammation, or an underlying disease[23, 24], respectively. Altogether, analysis of the goat blood characteristics/chemistry demonstrated that repeated AP procedures did not adversely affect the immediate or long-term health of these goats.

Intravenous jugular catheter vibration was sometimes observed with both venous draw and return cycles during plasmapheresis sessions. We suspected the vibrations were due to the diameter and length of the 16 gauge catheters being used. Switching from 16 gauge, 3 inch catheter to a 14 gauge, 1.5 inch catheter helped to mitigate this issue. In future studies, we will evaluate placement of indwelling IV jugular catheters. This may help to eliminate vibrations and decrease the amount of time required for repeated jugular IV catheter placement, thereby reducing the likelihood of trauma to jugular veins with repeated sampling.

This study shows improvements over the previous influenza inoculation and plasma collection procedures, by reducing the number of animals required and time needed to produce large volume of hyperimmune antisera for WHO HI reagent kits. In addition, goat antibodies showed greater specificity and comparable reactivity to influenza antigens, as compared to sheep antibodies (data not shown). Based on this work, we identified 48 hours as the optimal AP collection frequency and showed that we can safely collect 2000 mL of highly reactive plasma over 6 days. This amount of plasma is sufficient to rapidly produce sufficient HI surveillance kits to meet global influenza surveillance demand. This capability is critically important for global influenza surveillance since novel influenza viruses can emerge rapidly and spread globally in a matter of weeks[25], requiring the immediate manufacture of new influenza reagents. Altogether, this study demonstrates the importance of studying animal models to improve influenza surveillance and pandemic preparedness. Moreover, this highlights the need for further evaluation of goat models to reduce WHO kit manufacturing time and to improve the specificity of antisera reagents. Additionally we provide evidence that our model is adaptable for other research that requires rapid collection of large amounts of high quality plasma from goats.

## Abbreviations

APU: Automated Plasmapheresis Unit
AP: Automated Plasmapheresis
